# Chorea Associated with JAK2^V617F^ ‐Positive Essential Thrombocythemia

**DOI:** 10.1002/mdc3.13567

**Published:** 2022-09-30

**Authors:** Alessandra Calculli, Sebastiano Arceri, Antonio Pisani

**Affiliations:** ^1^ Department of Brain and Behavioral Sciences University of Pavia Pavia Italy; ^2^ IRCCS Mondino Foundation Pavia Italy

**Keywords:** chorea, essential thrombocythemia, Jak2, hyperkinetic disorders

It is with great interest that we read the article by Kutty and colleagues^1^ on chorea in patients with JAK2^V617F^‐mutated essential thrombocythemia (ET). As pointed out by the authors, the occurrence of chorea has rarely been reported, and its pathogenesis remains controversial. We would like to add to this discussion by describing a 61‐year‐old woman with a 3‐month history of slowly progressive chorea involving oromandibular district, left upper limb, and ipsilateral foot and toes. Five years before, the patient was diagnosed with JAK2^V617F^‐mutation ET and treated with hydroxyurea (500 mg once‐daily, OD). The subject autonomously interrupted the treatment right before symptoms onset. There was no history of other medical conditions or exposure to neuroleptic medication and no familiarity for neurological diseases. At admission to our department, her blood cell count showed platelet 816 × 10^3/μL, and brain magnetic resonance imaging (MRI) revealed no evidence of acute lesions but it did show a chronic vascular infarction in the right caudate nucleus. An autoantibodies panel, including ENA, ANA, ANCA, anti‐gliadin, anti‐cardiolipin, anti‐beta‐2‐glycoprotein and LAC, was negative. An electroencephalogram detected non‐specific slow‐wave activity, and genetic testing for Huntington's disease was negative.

During hospitalization, hydroxyurea was reintroduced at 1000 mg OD and tetrabenazine (12.5 mg twice‐daily, TD) was started, observing substantial clinical improvement. At the 6‐month follow‐up, her neurological examination was normal, and the platelet count normalized. Three months later, tetrabenazine was gradually withdrawn. However, after 4 to 5 weeks, choreic movements reappeared in the same districts, triggered by walking. Tetrabenazine treatment was then reintroduced, with beneficial effects.

Our case shares some similarities with the one described.^1^ In both cases, choreic movements coincided with deterioration of hematological parameters, which in our case was determined by voluntary withdrawal of hydroxyurea. Although we found a chronic ischemic lesion in the MRI, in a chorea of purely vascular nature, we would have expected a more acute presentation as well as a gradual resolution of symptoms, whereas similarly to the case reported,^1^ we also observed a slow, subacute onset of chorea, and, conversely, its gradual progression. Additionally, in our case, tetrabenazine interruption was followed shortly by symptom re‐emergence.

As proposed by the Koya Kutty et al.,^1^ blood hyperviscosity may not be sufficient to justify the development of chorea. In line with this view are the unusual symptom presentations and progression. An altered metabolic turnover of dopamine in the basal ganglia might cause adaptive changes in local transmitter content.^1^, ^2^ This hypothesis would fit with the prompt response to tetrabenazine.

Recently, a role for JAK2 in neuroinflammation has been proposed. JAK2 is member of a family of tyrosine kinases coupled to multiple signaling pathways. In particular, JAK2‐STAT3 pathway is required for astrocyte reactivity, and its inhibition reduces the astroglial response to quinolinic‐acid‐induced striatal lesions supporting its neuroprotective role,^3^, ^4^ which is expressed in striatal neurons. The JAK2^−V617F^ mutation causes the loss of its auto‐inhibitory activity, resulting in astrogliosis and inflammation. We postulate that local persistence of proinflammatory cytokines could impair striatal GABAergic signaling, generating choreic manifestations.^5^ Overall, it seems plausible that more than multiple pathophysiological factors concur in causing the appearance of chorea in these patients.

## Author Roles

(1) Research project: A. Conception, B. Organization, C. Execution; (2) Data Analysis: A. Design, B. Execution, C. Review and Critique; (3) Manuscript Preparation: A. Writing of the First Draft, B. Review and Critique.

A.C.: 1C, 3A.

S.A.: 1B, 2B.

A.P.: 1A, 2A, 2C, 3B.

## Disclosures

### Funding Sources and Conflicts of Interest

No specific funding was received for this work. The authors declare that there are no conflicts of interest relevant to this work.

### Financial Disclosures for the Previous 12 Months

The authors declare that there are no conflicts of interest relevant to this work.

### Ethical Compliance Statement

We confirm that we have read the Journal's position on issues involved in ethical publication and affirm that this work is consistent with those guidelines. Informed patient consent was verbally obtained. The authors confirm that the approval of an institutional review board was not required for this work.
